# Machine learning strategies for predicting pediatric suicidal behaviors in a Brazilian emergency setting

**DOI:** 10.3389/frai.2026.1662264

**Published:** 2026-02-18

**Authors:** Isis F. Carvalho, Ana Paula Couto da Silva, Anisio M. Lacerda, Wagner Meira, Danilo Bastos Bispo Ferreira, Lys Malloy Diniz, Alexandre Luiz de Oliveira Serpa, Maria Carolina Lobato Machado, Marco A. Romano-Silva, Debora Marques de Miranda, Gisele Lobo Pappa

**Affiliations:** 1 Departamento de Ciência da Computação, Universidade Federal de Minas Gerais, Belo Horizonte, MG, Brazil; 2 Faculdade de Medicina, Universidade Federal de Minas Gerais, Belo Horizonte, MG, Brazil; 3 PPG em Ciências do Desenvolvimento Humano, Universidade Presbiteriana Mackenzie, São Paulo, SP, Brazil; 4 Shanghai Mental Health Centre, Shanghai, China; 5 Centro de Tecnologia e Medicina Molecular, Faculdade de Medicina, Universidade Federal de Minas Gerais, Belo Horizonte, MG, Brazil

**Keywords:** adolescents, children, prediction, risk features, self-harm, suicide attempt, suicide ideation

## Abstract

**Background:**

Suicide is a leading cause of death worldwide, yet its prediction remains a challenge. This difficulty arises not only because suicidal behavior is a rare event in the general population, leading to significant class imbalance in datasets, but also due to its complex, multi-causal nature involving a non-linear interplay of sociodemographic and clinical factors. Furthermore, while the majority of suicides occur in middle-income countries, there is a lack of predictive models tailored to these specific social contexts. This study evaluates machine learning strategies in an enriched clinical setting: a pediatric psychiatric emergency center in Brazil.

**Methods:**

We analyzed a comprehensive database of 2,365 youth seeking emergency care. We benchmarked three machine learning algorithms, namely Logistic Regression, Random Forest, and XGBoost, to predict three outcomes: self-harm, suicidal ideation, and suicide attempts. To address class imbalance, we applied oversampling techniques to the training data. We also used SHapley Additive exPlanations (SHAP) values to quantify each feature's contribution to the predictions.

**Findings and interpretation:**

In this setting, suicide-related behaviors represented 28.7% of the clinical demand. The Random Forest model combined with oversampling was the most effective strategy, achieving sensitivities of 78.04% for suicidal ideation, 71.18% for suicide attempts, and 69.37% for self-harm. Specificity remained consistently above 75%. SHAP value analysis revealed that social determinants were critical predictors, highlighting that social conditions in middle-income populations introduce unique variables that significantly influence suicidal risk. While accuracy for suicide attempts remained a challenge, SHAP provided clear clinical insights into the drivers of risk.

**Conclusions:**

Machine learning, specifically Random Forest models together with oversampling and SHAP, demonstrates strong potential for identifying suicidal risk in pediatric emergency settings. By integrating clinical data with social determinants, these models provide a transparent and scalable strategy for early identification in regions with limited specialized psychiatric resources.

## 1 Introduction

Suicide claims a life every 40 s globally, and each loss impacts 60–135 people (Knipe et al., 2022). In 2020, a meta-analysis reported a suicide rate of 4.9/100,000 and a pooled rate of 17% for suicidal ideation among adolescents (Bersia et al., 2022). In developed countries, suicide stands as the foremost cause of death among children and adolescents (Knipe et al., 2022). The often underestimated burden of suicide and related behaviors weighs heavily on low- and middle-income countries (LMICs) (Knipe et al., 2022; Naghavi, 2019), which account for 75% of suicides worldwide. Hence, efforts to understand this phenomenon in these nations remain necessary, as few studies have examined them (Klonsky et al., 2016; Robinson et al., 2018). The differences in suicide rates in LMICs are mainly attributed to economic, social and cultural features, such as access to social support systems or mental health care (Miladinov, 2023).

Self-harm occurs at a 20 times higher frequency than suicide, and often precedes suicide attempts (Mars et al., 2019a). Both engaging in self-harm and being exposed to it are prominent predictors of suicide attempts (Mars et al., 2019a). In LMICs, the incidence of suicide planning and attempts among adolescents, especially girls aged 15–17, is approximately 17% (Uddin et al., 2019). Given the profound individual, societal and household impact of child and adolescent deaths, it is imperative to investigate the factors leading to this condition.

Multiple machine learning (ML) models have been proposed to predict suicide (Harris et al., 2019a). However, predicting suicide is a challenge even for proficient psychiatrists and mental health specialists. Understanding the interplay between social and environmental factors and mental health is crucial, as social factors often contribute to risk (Robinson et al., 2018; Pollock, 2019; Navarro et al., 2021). This holds particular significance in countries such as Brazil, where inequality and social shortcomings can exacerbate psychiatric risks (Glenn et al., 2020; Orellana et al., 2020).

Robust predictors for suicide-related behaviors offer a pathway to intervene in individuals' behavior or monitor specific variables. (Harris et al. 2019a) conducted a systematic review of risk assessment tools for predicting adolescent suicide (Harris et al., 2019b). They evaluated ten risk assessment tools used in the US and the UK and found that none of them could effectively predict suicidal behaviors. This reveals the complexity of the task, a matter that warrants significant attention given its widespread impact (Harris et al., 2019a,b).

In a population-based longitudinal study of Scottish young adults aged 18–34 (van Mens et al., 2020), the prediction of suicidal behaviors using traditional ML techniques found that algorithms based on decision trees outperformed regular logistic regression, achieving a sensitivity of 0.47 and a specificity of 0.91 for suicide attempt prediction. When comparing short- and long-term predictions of suicide risk using longitudinal data from structured electronic health records at the Connecticut Children's Medical Center, (Su et al. 2020) developed a model with sensitivity 0.62 and specificity 0.90; their model exhibited superior performance for shorter prediction windows. A model derived from a Brazilian cohort revealed a heightened risk of depression among individuals who are female, socially isolated, non-white, involved in drug use and conflicts, experiencing academic difficulties, and victims of maltreatment (Orellana et al., 2020).

Here, we propose models to predict self-harm, suicidal ideation and suicidal attempts using an enriched dataset from the regional emergency unit of the Psychiatric Emergency Center for Children and Adolescents (CEPAI-FHEMI), in Brazil. This facility provides urgent psychiatric care, primarily assisting individuals reporting depression, substance use, and symptoms of agitation and aggressiveness, as detailed in (Lobato Machado et al. 2022). This sample had more frequent suicidal behaviors than the general population, which may facilitate the identification of risks to the outcome (Robinson et al., 2018).

## 2 Methods

### 2.1 Ethics approval

This study was approved by the institutional review board (IRB) of the participating institution (COEP Ciencias Médicas). All procedures followed the ethical standards for human subjects research, and a waiver of informed consent was granted due to the retrospective design, in accordance with national regulations. The original data follows the rules from the medical records data storage, which must be retrieved only for patient benefit and must be safely stored for 20 years. Data was anonymized by removing names, addresses, document IDs, and other sensitive information. We report only aggregated patient statistics to avoid any risk of reidentification.

### 2.2 Dataset

We conducted a retrospective observational study with children and adolescents receiving emergency psychiatric care. The dataset comprises 2,365 health records of patients admitted to CEPAI-FHEMIG, some with multiple admissions, resulting in 1,720 unique patients (Lobato Machado et al., 2022). The data was collected from June 2017 to May 2018, and the subjects' ages range from 1 to 18 years. The dataset includes 27 sociodemographic features, such as race, gender, place of birth, household geographical location, school situation, living situation, and 123 clinical features, encompassing details such as reasons for seeking psychiatric assistance, family history of mental disorders, substance abuse information, psychiatric diagnoses received after treatment at the facility, neuro-psychomotor development delays, and previous traumatic events, among other relevant aspects.

We excluded 75 admissions where patients: (i) had no information at all (5); (ii) had only personal information (37); (iii) had no information about the motivations for looking for help or diagnoses (31); (iv) had not completed the screening stages at the center (1); or (v) had missing age information (1). Regarding the features, 12 of 110 contained textual information (e.g., a written reason for the patient leaving school). On average, more than 87% of the data in these 12 features were missing, and therefore these features were discarded. The original dataset also contained 21 features related to suicidal behaviors, which were, unfortunately, mostly missing (more than 80% of instances were missing) and were therefore excluded due to the high risk of data imputation in this context.

With the assistance of psychiatry professionals, out of the remaining 77 features, 57 were considered more informative for the tasks at hand. From the selected features, 33 were categorical. These categorical features were binarized, yielding 21 new binary features (e.g., whether the patient had depression, schizophrenia, or learning difficulties). The final dataset had 2,289 admissions for 1,687 unique patients (1,071 male and 616 female), with each admission described by 154 features.

Three target values—self-harm, suicidal ideation, and suicidal attempt—were extracted from the patient's motivation for seeking help, which was the prediction target. In this scenario, we had 337 out of 2,291 cases of self-harm (14.71%), 309 cases of suicide ideation (13.48%) and 323 positive suicide attempts (14.09%).

Self-harm was operationally defined as any non-suicidal manifestation of self-poisoning or self-injury lacking the explicit intent to induce mortality. Suicide ideation was identified in instances where individuals self-reported contemplation or strategic planning associated with suicidal tendencies. Concurrently, a suicide attempt was characterized as a deliberate act of self-poisoning or self-injury with the explicit aim of inducing mortality.

### 2.3 Methodology

To build prediction models for all outcomes, the classic methodology for handling data was followed. The first step was data preparation, as reported in the previous section. Next, the prepared datasets were used to train ML models, and oversampling was used to address class imbalance. As one of our main objectives is to interpret the contribution of each predictor to the model's decisions, we also employed SHapley Additive exPlanations (SHAP) (Lundberg and Lee, 2017) to understand predictions better. Each of these steps is detailed next.

The three ML models used in our experiments were Logistic Regression (LR), Random Forest (RF), and eXtreme Gradient Boosting (XGB) (Zaki and Meira, 2014). LR was chosen for its interpretability, whereas RF and XGB, as tree-based models, excel at capturing non-linear relationships and may facilitate the emergence of interpretable models. The experiments were conducted using the Scikit-learn (Pedregosa et al., 2011) and XGBoost libraries (Chen and Guestrin, 2016). A detailed description of these algorithms can be found in (Bishop 2006).

Given the imbalanced nature of the data, instances with suicidal behaviors were oversampled to compose 30% of the training set. Oversampling is a well-known technique that alters the data distribution in the training set to improve predictive performance by increasing the number of instances of the rarer class.

The models were trained on two distinct feature sets. The first set incorporates all available features and is denoted as “All Features” in the results table. The second set, referred to as “Feature Selection,” was generated using pre-selected features based on expert knowledge of the specialist who curated the dataset. It comprises features such as motivations for seeking help, diagnoses during medical hospitalization, whether it is the first-time admission to CEPAI-FHEMIG, and personal data, including gender, age, and the number of people in the household. We are aware that this type of selection can bias the model, and, for that reason, experiments with the full set of available features were also performed, and their results were compared with those obtained using only the features selected by the specialist.

During model training, the target variables were excluded from the set of predictors. Considering the relationship between suicide ideation and suicide attempt, ideation was used as a predictor for suicide attempt. Still, the suicide attempt was removed from the data used for suicide ideation prediction. For the self-harm prediction task, both suicidal ideation and suicide attempts were used as predictors, in line with previous research (Mars et al., 2019b).

Each model generated provided feature importance scores for the predictors, enabling us to compare the most significant features across classification tasks. Additionally, SHAP values were generated (Lundberg and Lee, 2017). SHAP is an approach rooted in game theory and frequently used to explain the output of ML models. It reveals the extent to which each feature contributes to the target feature. The interpretation of SHAP is akin to feature importance, but SHAP goes further by indicating whether each feature has a positive or negative relationship with the predicted value. It allows a richer understanding of feature impact, since it allows us to identify the most influential predictors associated with increased risk of suicide-related behaviors.

### 2.4 Experimental setup

To assess model performance, we used a stratified 10-fold cross-validation strategy, following the methodology detailed in (Bishop 2006). To prevent data leakage, we employed patient-level grouping: all admissions associated with a single individual were restricted to a single fold, ensuring the model was never tested on data from a patient it had already seen during training. For each iteration, the dataset was partitioned into training (eight folds), validation (one fold), and independent test sets (one fold). The validation set was used exclusively for hyperparameter optimization, whereas the test set was used only to assess model generalization. It is important to note that, in experiments using oversampling, the test set retained the original data distribution.

The performance of the models was assessed using four metrics:

1. Area under the receiver operating characteristic curve (AUC): assesses the model's performance across all classification thresholds of the true positive (TP) rate and false positive (FP) rate, providing a comprehensive view of the model's ability to discriminate between classes.
2. Positive predictive value (PPV): proportion of correctly predicted positive instances out of all predicted positive instances, offering insights into the accuracy of positive predictions.
3. Sensitivity: proportion of actual positive instances correctly identified by the classifier, indicating how well the model captures TP cases.
4. Specificity: proportion of actual negative instances correctly identified by the classifier, indicating how well the model captures true negative (TN) cases.

These metrics collectively provide a comprehensive evaluation of the model's performance on classification tasks. However, in the context of this paper, the sensitivity is considered more important than the other metrics, because the impact of not identifying a positive case of suicide attempt (or ideation, or self-harm) is considered more serious than misclassifying a negative case. This metric will be used to evaluate the models' performance relative to one another.

Regarding hyperparameter tuning, for XGB, a grid search was performed to identify a near-optimal parameter combination. This search encompassed four parameters: (1) the number of boosting iterations, (2) the ratio of features used to train a tree, (3) the maximum tree depth, and (4) the ratio of training instances in a subsample.

For RF, five parameters were assessed: (1) number of trees in the forest, (2) minimum number of samples required to split an internal node, (3) maximum depth of the tree, (4) minimum number of samples required to be at a leaf node and (5) whether bootstrap samples are used when building trees. Due to the large number of possible parameter combinations, an initial random search was conducted over the hyperparameter space. Subsequently, the best values identified in this search were evaluated using a grid search to determine the best parameter configuration. Table 1 presents the best parameter values found for Random Forest in the three scenarios investigated.

**Table 1:** Best parameter configurations returned by the grid-search for the Random Forest algorithm.

	**Model output**		
**Parameters**	**Suicide attempt**	**Suicide ideation**	**Self-harm**
Number of trees	10	10	100
Max tree depth	None	None	None
Min. number of split samples	2	4	1
Min. number of leaf samples	10	2	2
Use of bootstrap	True	True	True

Once the parameters for both RF and XGB models were determined, further tuning involved adjusting their classification thresholds, which determine whether an example is classified as positive or negative (default value is 0.5), using the validation set. This experiment was also performed for LR. In this scenario, we balanced sensitivity and specificity using the F1 score, which is the harmonic mean of PPV and sensitivity. After that, the models were evaluated on the test sets.

The three models underwent testing with both the complete set of attributes and the reduced set of features (“Feature Selection”). Both experiments were conducted with and without oversampling.

## 3 Results

### 3.1 Descriptive results

**Figure 1:**
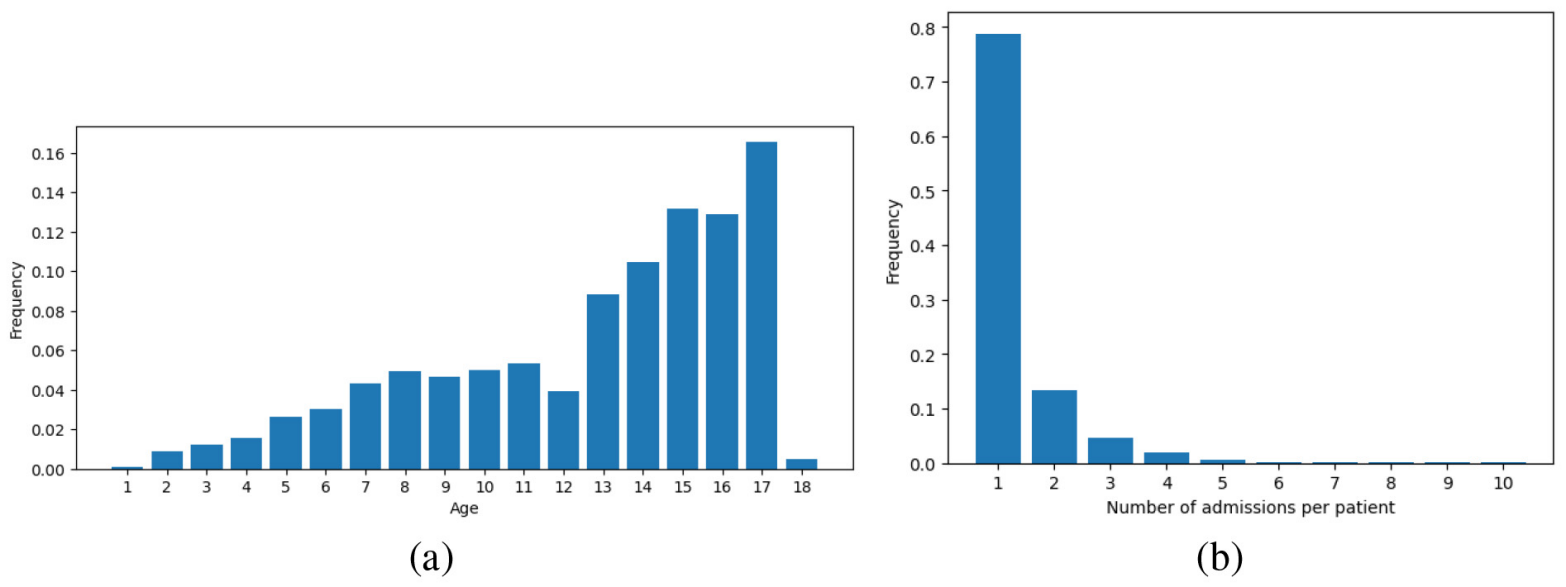
Statistics of the dataset. **(a)** Frequency of children searching for care in child psychiatry facilities; **(b)**Frequency of admissions for the same patient.

**Figure 2:**
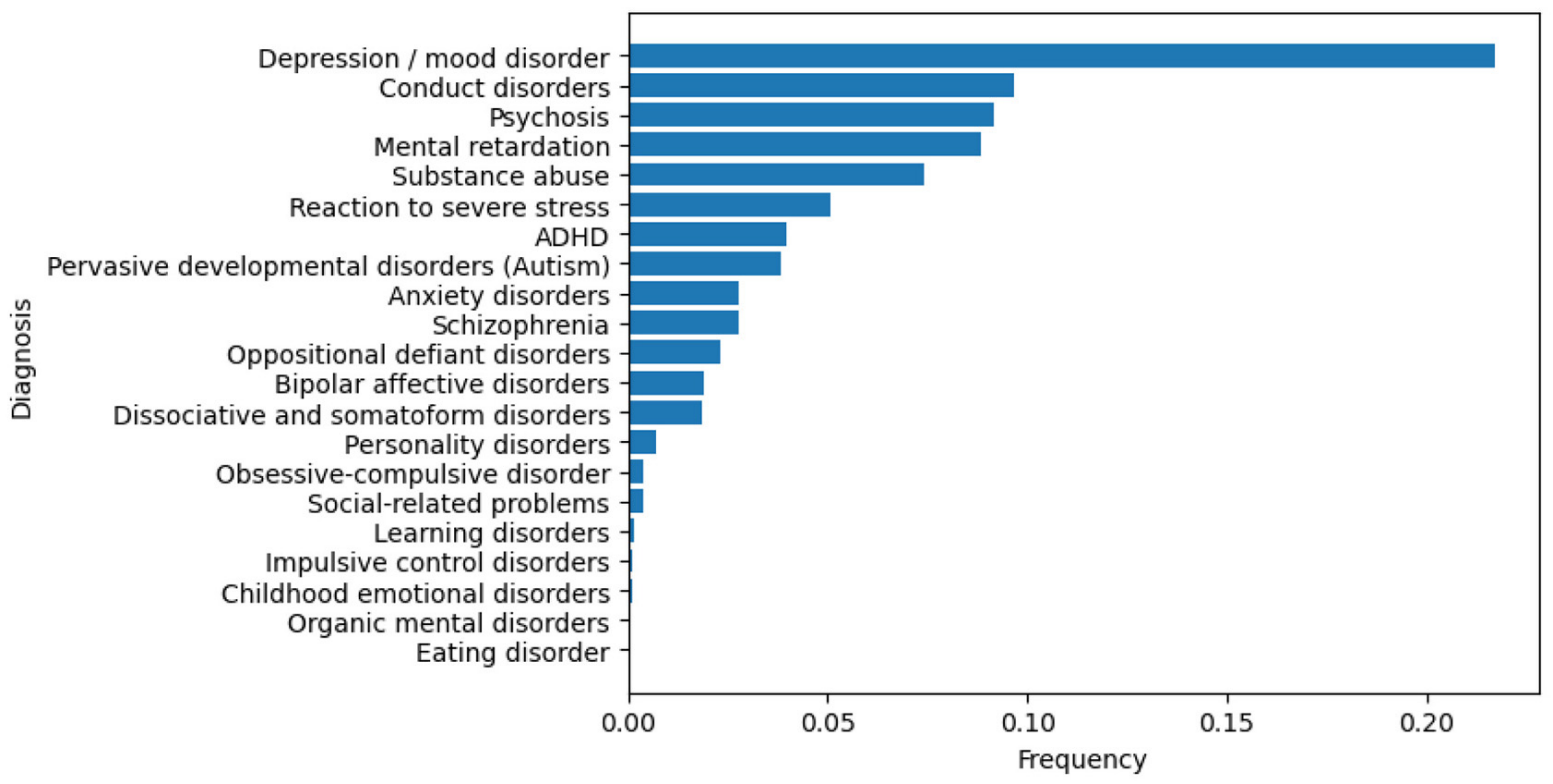
Frequency of diagnosis from admission in the psychiatric facility.

Figure 1 shows the basic statistics of the dataset. As observed in Figure 1a, most patients (~62%) were between 13 and 18 years old. Figure 1b shows the number of admissions to CEPAI-FHEMIG. Although most of the patients were admitted only once, 21% of the individuals were admitted at least twice in the studied period, revealing an incidence of repeated suicide behaviors, in line with the literature. Figure 2 illustrates the distribution of diagnoses given by the psychiatric professional (after assessment). Depression and general mood disorders were diagnosed in 21,69% of all admissions, being the most common diagnosis.

Table 2 presents descriptive statistics for a subset of features, including motives that led the family to seek help and other dependent features considered relevant to the model, using suicide attempt as the dependent variable. The most common reasons were related to agitation, aggressiveness, irritability and learning difficulties, which are not traditionally associated with suicidal behaviors. Factors commonly related to suicidal behavior include depression, self-harm, anxiety, and self-aggressiveness, which were present in 19,07%, 14,7%, 14,36% and 5,28% of the admissions registered, respectively, and follow the distribution in the table.

**Table 2:** Descriptive statistics of a subset of predictive variables, including motives to seek help, considering suicide attempt as the dependent variable.

**Independent variable**	**No suicide attempt (*N*= 1,966)**	**Suicide attempt (*N*=323)**	***p*-value**
Gender (male)	1,309 (66.6%)	129 (39.9%)	< 0.001
Gender (female)	657 (33.4%)	194 (60.1%)	< 0.001
Was hospitalized?	193 (9.8%)	81 (25.1%)	< 0.001
Age	12.6 ± 3.9	15.1 ± 1.9	< 0.001
Depression	288 (14.6%)	149 (46.1%)	< 0.001
Suicidal ideation	197 (10.0%)	112 (34.7%)	< 0.001
Self-harm	230 (11.7%)	107 (33.1%)	< 0.001
Substance abuse	324 (16.5%)	84 (26.0%)	–
Irritability	598 (30.4%)	74 (22.9%)	0.007
Aggressiveness	830 (42.2%)	69 (21.4%)	< 0.001
Insomnia	288 (14.6%)	63 (19.5%)	0.031
Isolation	236 (12.0%)	59 (18.3%)	0.003
Agitation	905 (46.0%)	49 (15.2%)	< 0.001
Anxiety	279 (14.2%)	47 (14.6%)	–
Hallucination	271 (13.8%)	46 (14.2%)	0.894
School difficulty	482 (24.5%)	40 (12.4%)	< 0.001
Disobedience	414 (21.1%)	38 (11.8%)	< 0.001
Mood swings	156 (7.9%)	21 (6.5%)	–
Attention difficulty	320 (16.3%)	15 (4.6%)	< 0.001
Delusion	182 (9.3%)	15 (4.6%)	0.008
Self-aggressiveness	108 (5.5%)	13 (4.0%)	0.338
Hypersexuality	47 (2.4%)	9 (2.8%)	0.816

*p*-values were obtained with a χ^2^ test for categorical attributes and a *t*-test for age, the only numerical attribute.

Suicide-related behaviors were among the motivations given by patients or their guardians to seek psychiatric care. As previously reported, the motivations were used to define the model's outcome variables. The dataset had 337 out of 2,291 cases of self-harm (14.71%), 309 cases of suicide ideation (13.48%) and 323 positive suicide attempts (14.09%).

### 3.2 Suicide behavior prediction results

Tables 3–5 present the results of the prediction models predicting suicide attempts, suicidal ideation, and self-harm, respectively. In the tables, the acronym OV denotes Oversampling, referring to models in which the data distribution was modified. The ratio of the outcome we want to predict corresponded to 30% of the training dataset. The RF model achieved the best overall performance across the four metrics for all three tasks. Oversampling does not affect the current sample's results, as the method's results with and without oversampling differ only in confidence intervals, which overlap. For suicide attempt and ideation prediction, the best-performing model used only the “Feature Selection” subset of features, achieving a sensitivity of 0.7118, specificity of 0.7592 and PPV of 0.3350 for suicide attempt prediction. For suicide ideation prediction, the method presented a sensitivity of 0.7804, specificity of 0.7763 and PPV of 0.3602. In the case of self-harm, the best model was trained using all available features, resulting in a sensitivity of 0.6937, specificity of 0.8177 and PPV of 0.4086.

**Table 3:** Suicide attempt prediction results for the three classifiers tested with and without oversampling (OV).

**Features**	**Models**	**AUC**	**PPV**	**Sensitivity**	**Specificity**
All features	LR	0.6582 ± 0.0628	0.3841 ± 0.0835	0.4350 ± 0.1494	0.8814 ± 0.0462
	LR OV	0.6956 ± 0.0678	0.3372 ± 0.0542	0.5837 ± 0.1614	0.8076 ± 0.0577
	RF	0.7338 ± 0.0659	0.3719 ± 0.0829	0.6583 ± 0.1067	0.8092 ± 0.0547
	RF OV	0.7379 ± 0.0543	0.3461 ± 0.0701	0.7047 ± 0.0990	0.7710 ± 0.0637
	XGB	0.6769 ± 0.0489	0.3256 ± 0.0565	0.5406 ± 0.1007	0.8132 ± 0.0394
	XGB OV	0.6989 ± 0.0786	0.3574 ± 0.0734	0.5682 ± 0.1610	0.8295 ± 0.0434
Feat. selection	LR	0.6808 ± 0.0523	0.4380 ± 0.0951	0.4665 ± 0.1135	0.8951 ± 0.0376
	LR OV	0.7264 ± 0.0644	0.3686 ± 0.0661	0.6428 ± 0.1504	0.8100 ± 0.0627
	RF	0.7225 ± 0.0430	0.3151 ± 0.0563	0.7055 ± 0.0932	0.7395 ± 0.0636
	RF OV	0.7355 ± 0.0430	0.3350 ± 0.0514	**0.7118** **±0.1079**	0.7592 ± 0.0627
	XGB	0.6812 ± 0.0553	0.3155 ± 0.0502	0.5628 ± 0.1272	0.7995 ± 0.0391
	XGB OV	0.7132 ± 0.0406	0.3012 ± 0.0241	0.6899 ± 0.0989	0.7364 ± 0.0366

In bold, there is the model and strategy with the best sensitivity.

**Table 4:** Suicide ideation prediction results for the three classifiers tested with and without oversampling (OV).

**Features**	**Models**	**AUC**	**PPV**	**Sensitivity**	**Specificity**
All features	LR	0.7053 ± 0.0406	0.4640 ± 0.0765	0.5075 ± 0.1040	0.9031 ± 0.0334
	LR OV	0.7418 ± 0.0445	0.4219 ± 0.0861	0.6274 ± 0.1252	0.8561 ± 0.0492
	RF	0.7599 ± 0.0434	0.4324 ± 0.0688	0.6625 ± 0.1212	0.8572 ± 0.0434
	RF OV	0.7819 ± 0.0540	0.4178 ± 0.0751	0.7287 ± 0.1373	0.8351 ± 0.0467
	XGB	0.7265 ± 0.0513	0.3596 ± 0.0683	0.6321 ± 0.1086	0.8210 ± 0.0384
	XGB OV	0.7782 ± 0.0304	0.3656 ± 0.0667	0.7755 ± 0.0896	0.7809 ± 0.0573
Feat. selection	LR	0.7250 ± 0.0415	0.4952 ± 0.1064	0.5449 ± 0.1096	0.9051 ± 0.0352
	LR OV	0.7397 ± 0.0364	0.4346 ± 0.0696	0.6099 ± 0.1046	0.8696 ± 0.0397
	RF	0.7557 ± 0.0340	0.3999 ± 0.0730	0.6781 ± 0.1020	0.8334 ± 0.0471
	RF OV	0.7783 ± 0.0363	0.3601 ± 0.0615	**0.7804** **±0.0954**	0.7763 ± 0.0525
	XGB	0.6991 ± 0.0466	0.3685 ± 0.0615	0.5477 ± 0.1001	0.8504 ± 0.0362
	XGB OV	0.7514 ± 0.0505	0.3929 ± 0.0643	0.6703 ± 0.1304	0.8324 ± 0.0475

In bold, there is the model and strategy with the best sensitivity.

**Table 5:** Self-harm prediction results for the three classifiers tested with and without oversampling (OV).

**Features**	**Models**	**AUC**	**PPV**	**Sensitivity**	**Specificity**
All features	LR	0.6989 ± 0.0511	0.4593 ± 0.0702	0.5072 ± 0.1348	0.8905 ± 0.0481
	LR OV	0.7315 ± 0.0582	0.4045 ± 0.0831	0.6348 ± 0.1394	0.8281 ± 0.0628
	RF	0.7333 ± 0.0367	0.4567 ± 0.0678	0.5955 ± 0.1039	0.8710 ± 0.0490
	RF OV	0.7557 ± 0.0340	0.4086 ± 0.0700	**0.6937** **±0.1075**	0.8177 ± 0.0600
	XGB	0.7198 ± 0.0288	0.3690 ± 0.0438	0.6305 ± 0.0873	0.8091 ± 0.0492
	XGB OV	0.7199 ± 0.0571	0.4693 ± 0.0692	0.5524 ± 0.1425	0.8874 ± 0.0447
Feat. selection	LR	0.7070 ± 0.0492	0.4906 ± 0.0809	0.5107 ± 0.1155	0.9033 ± 0.0391
	LR OV	0.7398 ± 0.0340	0.4112 ± 0.0640	0.6469 ± 0.0995	0.8327 ± 0.0505
	RF	0.7233 ± 0.0409	0.4594 ± 0.0817	0.5695 ± 0.1094	0.8771 ± 0.0483
	RF OV	0.7362 ± 0.0338	0.4065 ± 0.0608	0.6404 ± 0.0956	0.8321 ± 0.0498
	XGB	0.7155 ± 0.0405	0.3947 ± 0.0395	0.5902 ± 0.1084	0.8408 ± 0.0427
	XGB OV	0.7049 ± 0.0579	0.4877 ± 0.0454	0.5025 ± 0.1407	0.9073 ± 0.0299

In bold, there is the model and strategy with the best sensitivity.

In summary, a combination of sensitivity above 0.69 and specificity above 0.75 indicates accurate predictions, correctly identifying more than 69 out of 100 positive cases and more than 75 out of 100 negative cases. However, further studies are still needed to improve PPV. PPV indicates the likelihood that an individual with a positive suicide prediction is truly going to commit suicide. Although in this context false positives are more desirable than false negatives, more accurate models have better economic and social advantages and are more feasible. Improving the current model would require additional data, and we believe longitudinal data are key. We are pursuing efforts to obtain a more comprehensive dataset from CEPAI-FHEMIG. Given that we initially intend to use the system for triage, the burden of low PPV is smaller than that of a fully predictive system in operation. The worst-case scenario would prioritize an individual with low risk over one with higher risk.

Finally, it is important to say that we have analyzed the differences in the metrics regarding the training and test sets, and the model does not present any indication of overfitting. A cross-validation procedure was performed to obtain statistically meaningful results. However, we cannot guarantee the method will generalize to other contexts without further adaptation, and testing it in new scenarios is part of our future work.

### 3.3 SHAP values

**Figure 3:**
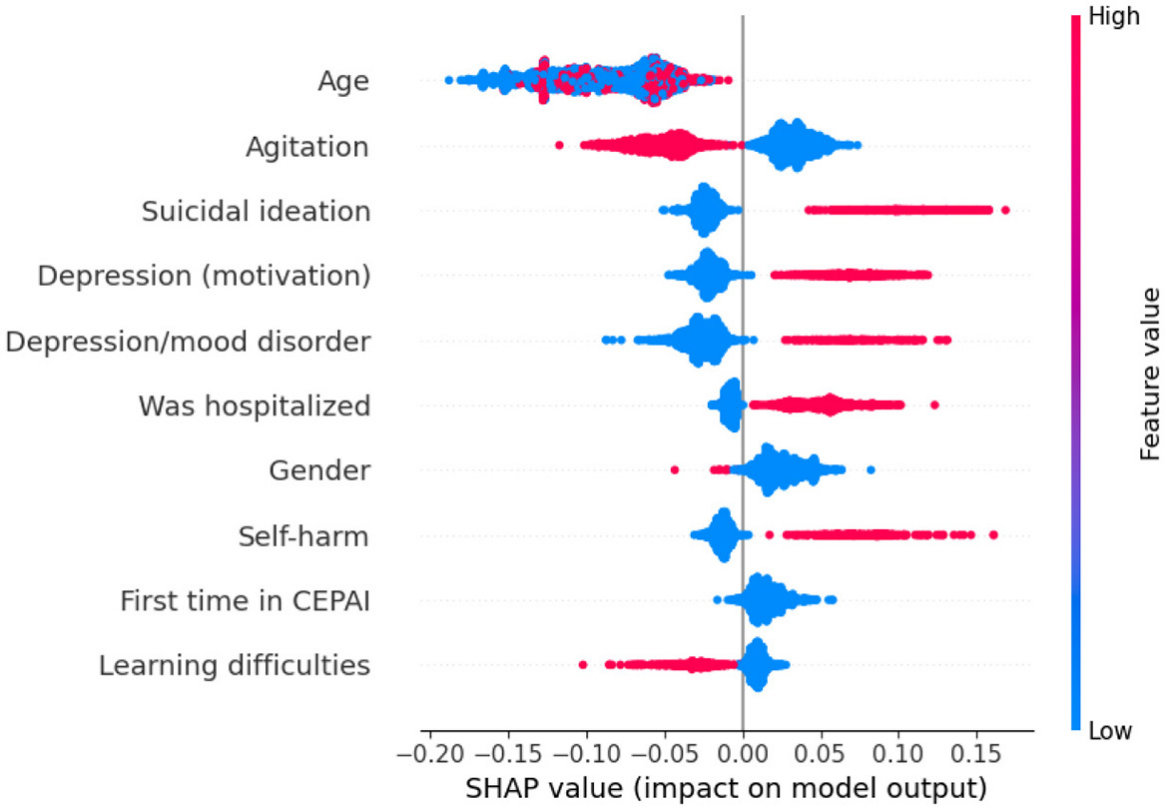
SHAP values for suicide attempt prediction. Each line represents a feature in decreasing order of importance, and the violin plot shows the impact of each feature in the model output. Features concentrated on the right side of the graph (positive SHAP) increase the prediction of suicide attempt, while features on the negative side decrease the prediction. Colors indicate whether a high (red) or low (blue) value of the feature is responsible for the increase/decrease in the prediction.

**Figure 4:**
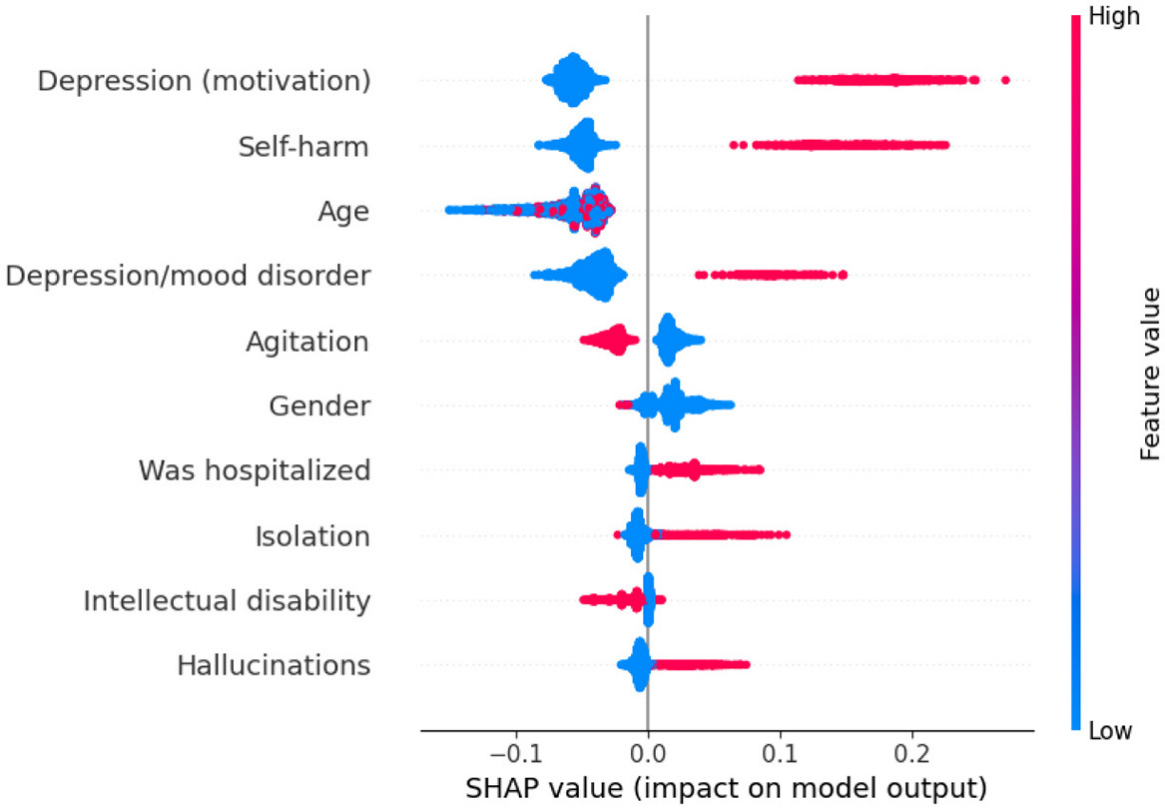
SHAP Values for Suicide Ideation Prediction. Each line represents a feature in decreasing order of importance, and the violin plot shows the impact of each feature in the model output. Features concentrated on the right side of the graph (positive SHAP) increase the prediction of suicide ideation, while features on the negative side decrease the prediction. Colors indicate whether a high (red) or low (blue) value of the feature is responsible for the increase/decrease in the prediction.

**Figure 5:**
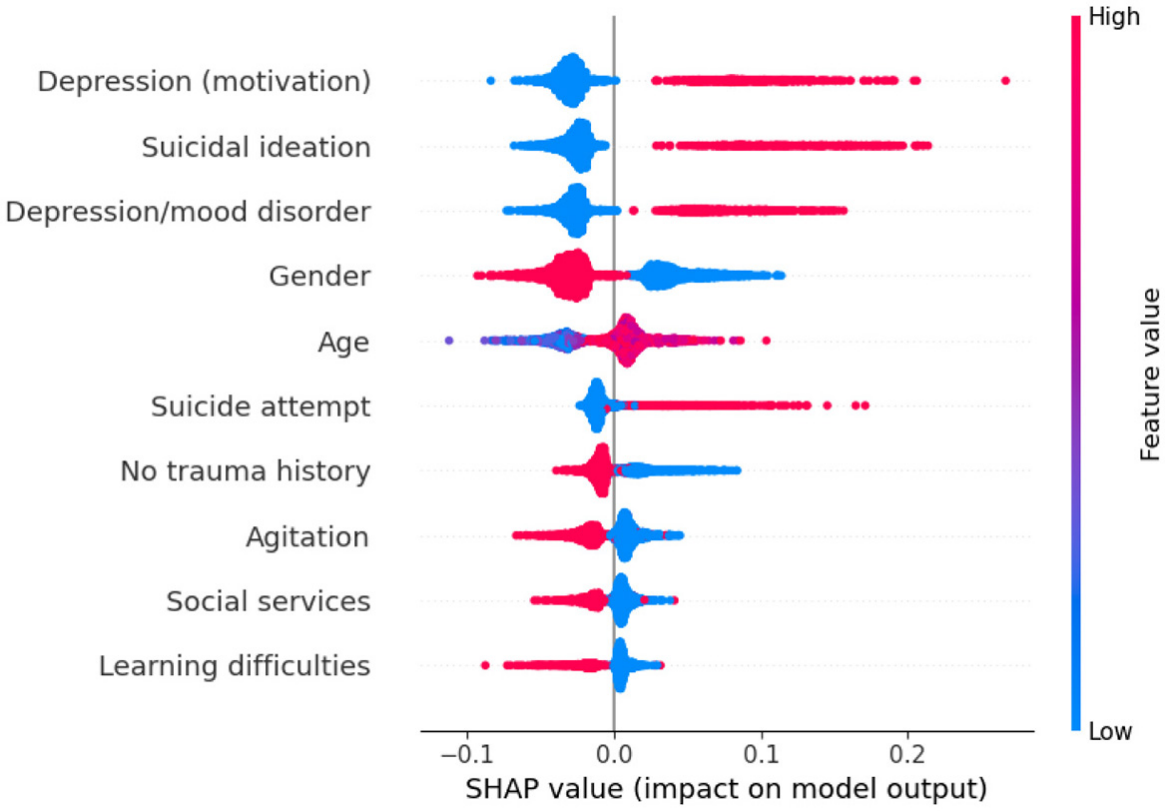
SHAP Values for Self-Harm Prediction. Each line represents a feature in decreasing order of importance, and the violin plot shows the impact of each feature in the model output. Features concentrated on the right side of the graph (positive SHAP) increase the prediction of self-harm, while features on the negative side decrease the prediction. Colors indicate whether a high (red) or low (blue) value of the feature is responsible for the increase/decrease in the prediction.

As we are interested in identifying the factors that most influence the prediction of suicidal behaviors, the impact of each feature on the model's output was assessed using SHAP values. Figures 3–5 depict these values for the three tasks. In these plots, the horizontal axis indicates whether a particular value is associated with a higher or lower prediction value. The colors denote whether that attribute presents a high (red) or low (blue) value. For this analysis, the gender feature is coded as 0 for female and 1 for male.

For example, higher values of “Agitation,” “Gender” (which means the patients are male), and “Learning difficulties” tend to produce lower predicted values for a suicide attempt (axis *x*), suggesting that male patients reporting agitation and learning difficulties are less likely to make a suicide attempt than the patients characterized by the opposite values of these binary variables (i.e., female patients, with no signs of agitation or learning difficulties). On the other hand, patients with depression, self-harm and suicidal ideation as motives for looking for help, who were hospitalized in CEPAI-FHEMIG and diagnosed with depression, are more likely to have a suicide attempt.

For suicide ideation (Figure 4), intellectual disability is linked to decreased risk, while isolation and hallucinations are associated with increased risk. As for the self-harm model (Figure 5), the relationships of gender and age with its prediction seem to be more regular, with older female patients being associated with a higher risk, while a patient with no trauma history using only CEPAI-FHEMIG's social services is linked to decreased risk, highlighting the distinct factors contributing to each outcome.

## 4 Discussion

The matter of children and adolescents requiring psychiatric care warrants attention. Roughly 1 in 5 children struggle with a mental, emotional, or behavioral disorder, yet fewer than 20% receive care from a mental health specialist (Vial and Almon, 2023). This lack of access to appropriate treatment is a pressing concern, particularly given that suicidal behavior, the most severe consequence of commonly untreated mental health issues, is a significant risk for this vulnerable population. In LMICs, accessing mental health services is even more difficult. Many individuals do not receive the care they need, and available services are often inefficient.

Suicidal behavior is a serious concern among adolescents, particularly during middle and late adolescence, with consequences that often persist into adulthood, especially for those who have previously attempted suicide. Research indicates that both short-term (6 months) and long-term (5 years) recurrence are common, increasing the risk among individuals who have been hospitalized for suicide attempts (Scoliers et al., 2009; Azcárate-Jiménez et al., 2019). Prevalence rates for suicidal ideation and attempts range from 14.3% to 22.6% and 4.6% to 15.8%, respectively (Van Meter et al., 2023). The transition from adolescence to young adulthood is associated with high healthcare costs and frequently results in interruptions in psychiatric care (Canaway et al., 2023). Late adolescence is the period of greatest risk for suicidal behavior, particularly during stressful times (Whalen et al., 2022; Orri et al., 2020). There is a pressing need to understand the predictors of suicidal behaviors to develop effective preventive interventions and ensure accessible, appropriate care for adolescents and young adults.

While ML excels at general classification and prediction tasks, predicting suicide behaviors remains a complex challenge. Suicide-related behaviors are influenced by numerous, interrelated factors that cannot be addressed with simple solutions. As noted earlier, a suicide attempt prediction model achieved a sensitivity of 0.47 and a specificity of 0.91 (van Mens et al., 2020). Although direct comparison is difficult due to differences in datasets, their study demonstrated higher specificity (0.76) but lower sensitivity (0.71) compared to ours. This underscores the critical importance of calibrating classifier thresholds. Adjusting these thresholds can significantly affect model performance, enabling tailored outcomes aligned with clinical priorities. Given the potential severity of missed cases, we prioritize increased sensitivity, while acknowledging that this often comes at the expense of specificity.

In sum, dealing with prediction tasks of suicidal behaviors is hard because (i) the data has an intrinsic bias—all patients presenting at the emergency had psychiatric symptoms; (ii) the suicide attempt itself is an unbalanced data; (iii) the dataset is limited to a low number of patients; (iv) mispredicting a high-risk patient has more serious consequences, given that it is a cost-sensitive classification task. As most health-related models, the one generated here would need to be adapted to a setting that resembles the prevalence of these events in the general population (Richter et al., 2025). We are currently looking for a new dataset to validate our findings.

Given the numerous predisposing conditions, the relatively low frequency of the tragic event, and the undesirable possibility of error, the models proposed in this study demonstrate acceptable accuracy and can serve as valuable screening tools. These models can aid health professionals by identifying patients at risk of suicidal behavior. They may function effectively as screening tools, prioritizing individuals with a high probability of suicide risk for prompt medical attention. Such measures that facilitate access could help minimize adverse outcomes. This approach is particularly crucial in middle-income scenarios where access to psychiatric facilities and treatment is insufficient for the multitude of people in deep need of care (Dalgalarrondo et al., 2023).

The unique context of addressing psychiatric cases in an emergency setting significantly differentiates our work. Patients in crisis alter the population-level expectation of rare events, making them more frequent. The emphasis put on identifying predictors of suicide-related behaviors allowed our models to pinpoint the most relevant factors for self-harm, suicidal ideation, and suicide attempts, permitting timely intervention. The potential of these predictor models lies in prioritizing higher-risk patients for immediate emergency care and identifying the risk factors involved in each case. Given the rarity of the outcome in high-risk populations (lower heterogeneity), there is a clear need to validate our model on data from other psychiatric centers to generalize the results (Varoquaux, 2018). Beyond the difficulties in predicting low-frequency events, another limitation of the study was the associative character of the risk features, which does not add causal knowledge that should be addressed at some point.

The consistent identification of the 10 most relevant features across models for predicting self-harm, suicidal ideation, and suicide attempts highlights the strong interconnection among these outcomes. When assessment resources are limited, key indicators of suicidal behavior become increasingly prominent, especially those linked to lack of treatment, inadequate care, and repeated psychiatric service utilization. Social factors, such as household size, may also shed light on vulnerability, particularly in LMICs, where these dynamics differ. While emergency settings provide a valuable source of cases for model training, they also introduce limitations, as findings may not generalize readily to other clinical contexts.

Depression and its symptoms consistently stand out as major predictors of suicide-related behaviors, as supported by previous research (Li, 2023; Jiang et al., 2021; Mubasyiroh et al., 2018). Given its substantial predictive value, depression should be prioritized as a key target for intervention. Early detection and effective treatment of depression could substantially reduce the risk of suicide-related outcomes. In addition, self-harm and suicidal ideation are vital warning signs that may indicate the potential for more severe developments. Addressing modifiable social determinants, integrated into risk screening, can further enhance the comprehensiveness of suicide prevention strategies. Limited access to mental health care is also strongly associated with increased risk, as individuals often seek help in emergency settings due to inadequate prior psychiatric support. These findings highlight the urgent need to improve access to mental health services and initiate timely interventions to prevent escalation and severe outcomes.

## 5 Conclusions

This study introduces a machine learning framework designed to predict suicidal behaviors, including suicidal ideation, self-harm, and suicide attempts, within a pediatric psychiatric emergency context. By focusing on a middle-income population in Brazil, this research addresses a significant geographic gap. Although most global suicides occur in LMICs, these settings remain significantly underrepresented in predictive modeling literature.

Our findings lead to several key conclusions regarding the intersection of artificial intelligence and youth mental health: The Random Forest model with oversampling achieved sensitivities ranging from 69% to 78%, demonstrating its clinical utility for identifying at-risk individuals who might otherwise be missed in overstretched emergency services. By using SHAP values, we moved from “black-box” predictions, showing social determinants are primary drivers of risk in middle-income contexts.

Ultimately, this study serves as a proof-of-concept for the deployment of AI-supported screening tools in psychiatric emergency departments. By integrating these models into triage workflows, we can shift from reactive crisis management to proactive, data-driven identification, potentially reducing suicide rates in vulnerable youth populations worldwide.

## Data Availability

The raw data supporting the conclusions of this article will be made available by the authors, without undue reservation.

## References

[B1] Azcárate-Jiménez L. López-Goñi J. Goñi-Sarriés A. Montes-Reula L. Portilla-Fernández A. Elorza-Pardo R. . (2019). Repeated suicide attempts: a follow-up study. Actas Esp. Psiquiatr. 47, 127–136. Available online at: https://actaspsiquiatria.es/index.php/actas/article/view/245 (Accessed January, 2026). 31461152

[B2] Bersia M. Koumantakis E. Berchialla P. Charrier L. Ricotti A. Grimaldi P. . (2022). Suicide spectrum among young people during the COVID-19 pandemic: a systematic review and meta-analysis. EClinicalMedicine 54:101705. doi: 10.1016/j.eclinm.2022.101705 36338787 PMC9621691

[B3] Bishop C. (2006). Pattern Recognition and Machine Learning. New York, NY: Springer.

[B4] Canaway A. Appleton R. van Bodegom L. Dieleman G. Franić T. Gerritsen S. . (2023). Healthcare costs for young people transitioning the boundary between child/adolescent and adult mental health services in seven European countries: results from the milestone study. BJPsych Open 9:e175. doi: 10.1192/bjo.2023.559 37749976 PMC10617498

[B5] Chen T. Guestrin C. (2016). “Xgboost: a scalable tree boosting system,” in Proceedings of the 22nd ACM SIGKDD International Conference on Knowledge Discovery and Data Mining (New York, NY: ACM), 785–794. doi: 10.1145/2939672.2939785

[B6] Dalgalarrondo P. Oda A. Onocko-Campos R. Banzato C. (2023). Challenges facing the psychiatric reform and mental health care in Brazil: critical unmet needs and prospects for better integrating the public and university sectors. SSM - Ment. Health 4:100262. doi: 10.1016/j.ssmmh.2023.100262

[B7] Glenn C. Kleiman E. Kellerman J. Pollak O. Cha C. Esposito E. . (2020). Annual research review: a meta-analytic review of worldwide suicide rates in adolescents. J. Child Psychol. Psychiatry 61, 294–308. doi: 10.1111/jcpp.13106 31373003

[B8] Harris I. Beese S. Moore D. (2019a). Predicting future self-harm or suicide in adolescents: a systematic review of risk assessment scales/tools. BMJ Open 9:e029311. doi: 10.1136/bmjopen-2019-029311 31494608 PMC6731844

[B9] Harris I. Beese S. Moore D. (2019b). Predicting repeated self-harm or suicide in adolescents and young adults using risk assessment scales/tools: a systematic review protocol. Syst. Rev. 8:87. doi: 10.1186/s13643-019-1007-7 30947743 PMC6449918

[B10] Jiang T. Nagy D. Rosellini A. Horváth-Puhó E. Keyes K. Lash T. . (2021). Suicide prediction among men and women with depression: a population-based study. J. Psychiatr. Res. 142, 275–282. doi: 10.1016/j.jpsychires.2021.08.003 34403969 PMC8456450

[B11] Klonsky E. May A. Saffer B. (2016). Suicide, suicide attempts, and suicidal ideation. Annu. Rev. Clin. Psychol. 12, 307–330. doi: 10.1146/annurev-clinpsy-021815-093204 26772209

[B12] Knipe D. Padmanathan P. Newton-Howes G. Chan L. Kapur N. (2022). Suicide and self-harm. Lancet 399, 1903–1916. doi: 10.1016/S0140-6736(22)00173-8 35512727

[B13] Li Y. (2023). Depression and suicide risk prediction based on machine learning models. J. Educ. Humanit. Soc. Sci. 15, 302–307. doi: 10.54097/ehss.v15i.9312

[B14] Lobato Machado M. Hibner M. Nogueira D. Rezende M. Bórem I. da Cunha L. . (2022). Irritability in an open-door pediatric psychiatric emergency service in a middle-income country. Neuropsychiatr. Enfance Adolesc. 70, 336–342. doi: 10.1016/j.neurenf.2022.05.007

[B15] Lundberg S. Lee S. (2017). “A unified approach to interpreting model predictions” in Paper Presented at the 31st Conference on Neural Information Processing Systems (NIPS 2017) (Long Beach, CA).

[B16] Mars B. Heron J. Klonsky E. Moran P. O'Connor R. Tilling K. . (2019a). Predictors of future suicide attempt among adolescents with suicidal thoughts or non-suicidal self-harm: a population-based birth cohort study. Lancet Psychiatry 6, 327–337. doi: 10.1016/S2215-0366(19)30030-6 30879972 PMC6494973

[B17] Mars B. Heron J. Klonsky E. Moran P. O'Connor R. Tilling K. . (2019b). What distinguishes adolescents with suicidal thoughts from those who have attempted suicide? A population-based birth cohort study. J. Child Psychol. Psychiatry 60, 91–99. doi: 10.1111/jcpp.12878 29492978 PMC6334515

[B18] Miladinov G. (2023). The economics of suicide: evidence from LMICS and HICS. J. Res. Soc. Sci. Humanit. 2, 9–25. doi: 10.56397/JRSSH.2023.06.02

[B19] Mubasyiroh R. Pradono J. Nurkhotimah E. Kusumawardani N. Idaiani S. (2018). Depression as a strong prediction of suicide risk. Glob. J. Health Sci. 10:52. doi: 10.5539/gjhs.v10n12p52

[B20] Naghavi M. (2019). Global, regional, and national burden of suicide mortality 1990 to 2016: systematic analysis for the global burden of disease study 2016. BMJ 364:l94. doi: 10.1136/bmj.l94 31339847 PMC6598639

[B21] Navarro M. Ouellet-Morin I. Geoffroy M. Boivin M. Tremblay R. Côté S. . (2021). Machine learning assessment of early life factors predicting suicide attempt in adolescence or young adulthood. JAMA Netw. Open 4:e211450. doi: 10.1001/jamanetworkopen.2021.1450 33710292 PMC7955274

[B22] Orellana J. Ribeiro M. Barbieri M. Saraiva M. C. Cardoso V. Bettiol H. . (2020). Mental disorders in adolescents, youth, and adults in the RPS birth cohort consortium (Ribeirão Preto, Pelotas and São Luís), Brazil. Cad. Saude Publica 36:e00154319. doi: 10.1590/0102-311x00154319 32022176

[B23] Orri M. Scardera S. Perret L. Bolanis D. Temcheff C. Séguin J. . (2020). Mental health problems and risk of suicidal ideation and attempts in adolescents. Pediatrics 146:e20193823. doi: 10.1542/peds.2019-3823 32513840

[B24] Pedregosa F. Varoquaux G. Gramfort A. Michel V. Thirion B. Grisel O. . (2011). Scikit-learn: machine learning in python. J. Mach. Learn. Res. 12, 2825–2830. Available online at: http://scikit-learn.sourceforge.net (Accessed January, 2026).

[B25] Pollock N. (2019). Place, the built environment, and means restriction in suicide prevention. Int. J. Environ. Res. Public Health, 16. doi: 10.3390/ijerph16224389 31717635 PMC6888187

[B26] Richter M. Emden D. Leenings R. Winter N. R. Mikolajczyk R. Massag J. . (2025). Generalizability of clinical prediction models in mental health. Mol. Psychiatry 30, 3632–3639. doi: 10.1038/s41380-025-02950-0 40108256 PMC12240828

[B27] Robinson J. Bailey E. Witt K. Stefanac N. Milner A. Currier D. . (2018). What works in youth suicide prevention? A systematic review and meta-analysis. EClinicalMedicine 4–5, 52–91. doi: 10.1016/j.eclinm.2018.10.004 31193651 PMC6537558

[B28] Scoliers G. Portzky G. van Heeringen K. Audenaert K. (2009). Sociodemographic and psychopathological risk factors for repetition of attempted suicide: a 5-year follow-up study. Arch. Suicide Res. 13, 201–213. doi: 10.1080/13811110902835130 19590995

[B29] Su C. Aseltine R. Doshi R. Chen K. Rogers S. Wang F. . (2020). Machine learning for suicide risk prediction in children and adolescents with electronic health records. Transl. Psychiatry 10:413. doi: 10.1038/s41398-020-01100-0 33243979 PMC7693189

[B30] Uddin R. Burton N. Maple M. Khan S. Khan A. (2019). Suicidal ideation, suicide planning, and suicide attempts among adolescents in 59 low-income and middle-income countries: a population-based study. Lancet Child Adolesc Health 3, 223–233. doi: 10.1016/S2352-4642(18)30403-6 30878117

[B31] van Mens K. de Schepper C. Wijnen B. Koldijk S. Schnack H. de Looff P. . (2020). Predicting future suicidal behaviour in young adults, with different machine learning techniques: a population-based longitudinal study. J. Affect. Disord. 271, 169–177. doi: 10.1016/j.jad.2020.03.081 32479313

[B32] Van Meter A. Knowles E. Mintz E. (2023). Systematic review and meta-analysis: international prevalence of suicidal ideation and attempt in youth. J. Am. Acad. Child Adolesc. Psychiatry 62, 973–986. doi: 10.1016/j.jaac.2022.07.867 36563876 PMC12702477

[B33] Varoquaux G. (2018). Cross-validation failure: small sample sizes lead to large error bars. Neuroimage 180, 68–77. doi: 10.1016/j.neuroimage.2017.06.061 28655633

[B34] Vial T. Almon A. (2023). Artificial intelligence in mental health therapy for children and adolescents. JAMA Pediatr. 177, 1251–1252. doi: 10.1001/jamapediatrics.2023.4212 37843842

[B35] Whalen D. Hennefield L. Elsayed N. Tillman R. Barch D. Luby J. . (2022). Trajectories of suicidal thoughts and behaviors from preschool through late adolescence. J. Am. Acad. Child Adolesc. Psychiatry 61, 676–685. doi: 10.1016/j.jaac.2021.08.020 34506928 PMC8898992

[B36] Zaki M. Meira W. (2014). Data Mining and Analysis: Fundamental Concepts and Algorithms. Cambridge, MA: Cambridge University Press. doi: 10.1017/CBO9780511810114

